# Supplementation with inulin-type fructans affects gut microbiota and attenuates some of the cardiometabolic benefits of a plant-based diet in individuals with overweight or obesity

**DOI:** 10.3389/fnut.2023.1108088

**Published:** 2023-04-25

**Authors:** Mona Adnan Aldubayan, Xiaotian Mao, Martin Frederik Laursen, Kristina Pigsborg, Lars H. Christensen, Henrik M. Roager, Dennis S. Nielsen, Mads Fiil Hjorth, Faidon Magkos

**Affiliations:** ^1^Department of Clinical Nutrition, College of Applied Medical Sciences, King Saud bin Abdulaziz University for Health Sciences, Riyadh, Saudi Arabia; ^2^Department of Nutrition, Exercise and Sports, Faculty of Science, University of Copenhagen, Copenhagen, Denmark; ^3^Department of Food Science, Faculty of Science, University of Copenhagen, Copenhagen, Denmark; ^4^National Food Institute, Technical University of Denmark, Kongens Lyngby, Denmark; ^5^Obesity and Nutritional Sciences, Novo Nordisk Foundation, Tuborg Havnevej, Hellerup, Denmark

**Keywords:** personalized nutrition, precision nutrition, microbiome, plant-based diet, inulin-type fructans, prebiotics, obesity, cardiometabolic health

## Abstract

**Background:**

The gut microbiota has emerged as a potential therapeutic target to improve the management of obesity and its comorbidities.

**Objective:**

We investigated the impact of a high fiber (∼38 g/d) plant-based diet, consumed *ad libitum*, with or without added inulin-type fructans (ITF), on the gut microbiota composition and cardiometabolic outcomes in subjects with obesity. We also tested if baseline *Prevotella/Bacteroides* (P/B) ratio predicts weight loss outcomes.

**Methods:**

This is a secondary exploratory analysis from the PREVENTOMICS study, in which 100 subjects (82 completers) aged 18–65 years with body mass index 27–40 kg/m^2^ were randomized to 10 weeks of double-blinded treatment with a personalized or a generic plant-based diet. Changes from baseline to end-of-trial in gut microbiota composition (16S rRNA gene amplicon sequencing), body composition, cardiometabolic health and inflammatory markers were evaluated in the whole cohort (*n* = 82), and also compared in the subgroup of subjects who were supplemented with an additional 20 g/d ITF-prebiotics (*n* = 21) or their controls (*n* = 22).

**Results:**

In response to the plant-based diet, all subjects lost weight (−3.2 [95% CI –3.9, −2.5] kg) and experienced significant improvements in body composition and cardiometabolic health indices. Addition of ITF to the plant-based diet reduced microbial diversity (Shannon index) and selectively increased *Bifidobacterium* and *Faecalibacterium* (*q* < 0.05). The change in the latter was significantly associated with higher values of insulin and HOMA-IR and lower HDL cholesterol. In addition, the LDL:HDL ratio and the concentrations of IL-10, MCP-1 and TNFα were significantly elevated in the ITF-subgroup. There was no relationship between baseline P/B ratio and changes in body weight (*r* = −0.07, *p* = 0.53).

**Conclusion:**

A plant-based diet consumed *ad libitum* modestly decreases body weight and has multiple health benefits in individuals with obesity. Addition of ITF-prebiotics on top this naturally fiber-rich background selectively changes gut microbiota composition and attenuates some of the realized cardiometabolic benefits.

**Clinical trial registration:**

[https://clinicaltrials.gov/ct2/show/NCT04590989], identifier [NCT04590989].

## Introduction

It is well-established that a dynamic population of microorganisms residing in the gastrointestinal tract—namely, the gut microbiota—partakes in regulating various host signaling pathways and physiological functions including energy homeostasis, glucose and lipid metabolism, and inflammatory responses (1–3). These processes have been shown to be partly mediated by the production of microbial metabolites such as short-chain fatty acids (SCFAs)—primarily acetate, propionate, and butyrate—from the fermentation of dietary fiber and resistant starch in the gut (1, 2). Disruption of intestinal microbiota function can alter its derived metabolites and byproducts, which may trigger a broad range of physiological responses linked to increased risk of developing metabolic diseases such as obesity and type 2 diabetes (T2D) (2, 3).

Diet is a key factor shaping the composition and function of gut microbiota (2, 4). Dietary patterns such as vegetarian or vegan diets and animal-based diets demonstrate distinct effects on gut microbiota composition (5, 6). Plant-based diets contain a variety of whole grains and are typically rich in fiber, while lower in saturated fat and protein as opposed to omnivorous diets (5, 7). Greater adherence to plant-based diets was reported to have favorable outcomes on body weight homeostasis and cardiometabolic heath (7–9) potentially and partly *via* promoting the preservation of a more diverse and stable ecosystem of beneficial bacteria residing in the gut (6). Different types of dietary fibers—depending on their chemical structure and physical properties—may stimulate the growth of specific taxa that express specific genes encoding enzymes required for their metabolism (4, 10). For example, inulin-type fructans (ITFs) are a type of fermentable dietary fibers known as prebiotics that induce beneficial health effects resulting from cross-feeding interactions between bifidobacteria and butyrate-producing bacteria (11). ITF-prebiotics, including fructo-oligosaccharides, oligofructose and inulin, are among the most investigated and well-established prebiotics, and have been shown to affect intestinal health and metabolism (11–14). However, there is a notable variation in the effects of dietary fiber on gut microbiota (15) and host health, which may be attributed to the unique microbial community that each individual harbors and its fiber-fermentation capacity (16, 17). This highly individualized response is opening the door to discover new potential microbiota-targeted therapeutics for the prevention and management of obesity and its metabolic comorbidities through precision nutrition (18). From this perspective, profiling the gut microbiome is emerging as a promising prognostic marker to predict response to dietary interventions (19). For instance, recent studies have found that individuals with high *Prevotella*-to-*Bacteroides* (P/B) ratio lose more weight than individuals with low P/B ratio when consuming a diet rich in whole grains and fiber (20–22), presumably due to the distinct fermentation responses dependent on *Prevotella* species (16, 19). Accordingly, in this secondary exploratory analysis from the PREVENTOMICS study (23, 24), the main objective was to investigate the impact of consuming an *ad libitum* high-fiber plant-based diet with or without the addition of isolated ITF-prebiotics on changes in gut microbiota in subjects with obesity, and explored associations with different metabolic health markers. In addition, we investigated whether weight loss can be predicted based on baseline P/B ratio as previously suggested.

## Materials and methods

### Study design

The PREVENTOMICS trial was a 10-week randomized, double-blind, placebo-controlled study conducted from October 2020 to June 2021 at the Department of Nutrition, Exercise and Sports (NEXS) at the University of Copenhagen, Denmark, according to the Declaration of Helsinki guidelines. The study was approved by the ethical committee of the Capital Region of Denmark (H-20029882), and the trial was registered at clinicaltrials.gov (NCT04590989). All participants signed an informed consent prior to inclusion.

**Figure 1:**
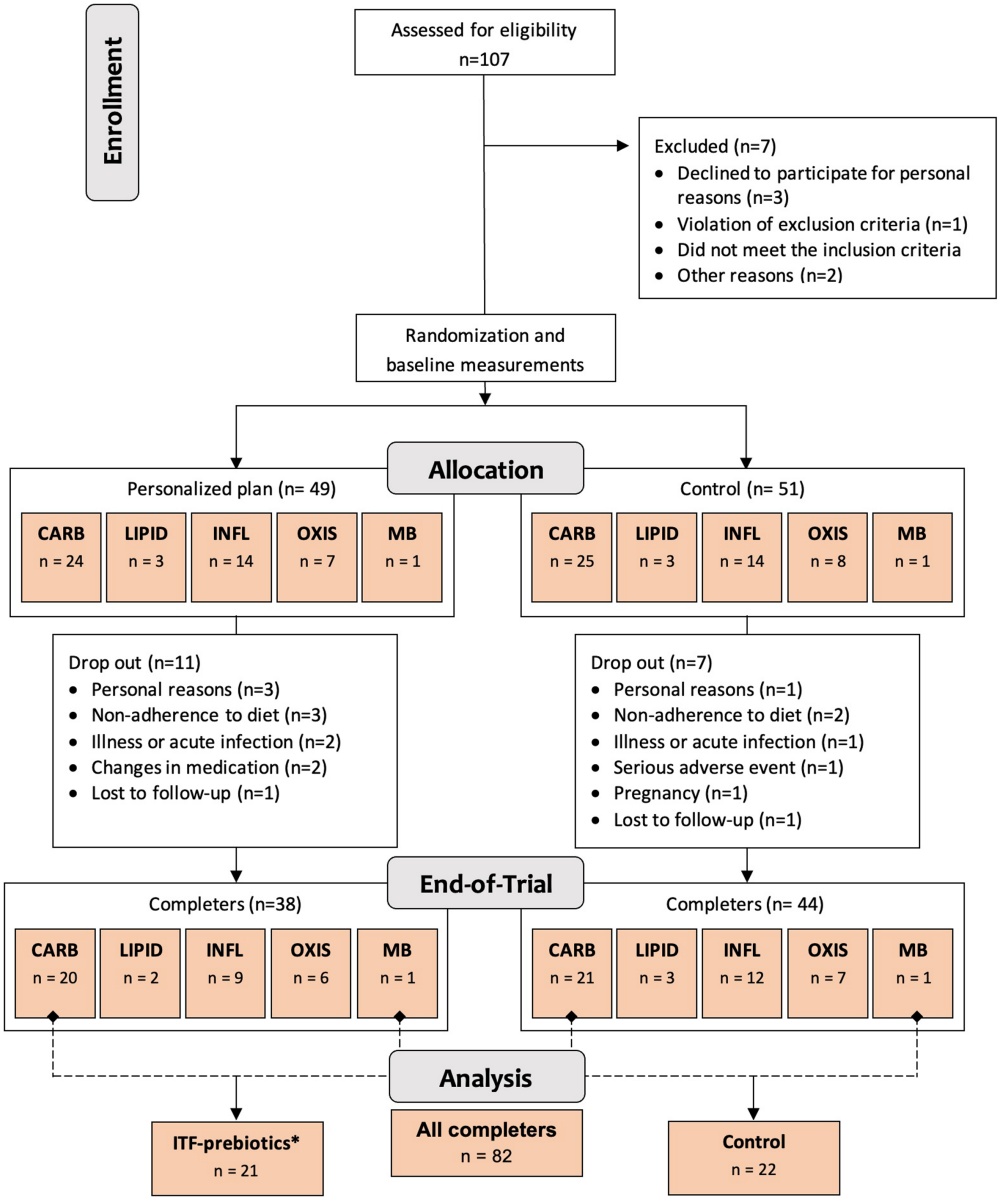
Study design and flowchart. Abbreviations: ITF, inulin-type fructans; CARB, carbohydrate cluster; LIPID, lipid cluster; INFL, inflammation cluster; OXIS, oxidative stress cluster; MB, microbiota cluster. ^*^Group receiving 20 g/d inulin + fructooligosaccharides.

One hundred generally healthy subjects (free from diagnosis of cancer and chronic diseases such as diabetes, cardiovascular, renal or liver disease (23)) aged 18–65 years, with body mass index ≥27 but <40 kg/m^2^ and elevated waist circumference (males >94 cm; females >80 cm), were randomly assigned in a 1:1 ratio stratified by cluster (oxidative stress; inflammation; carbohydrate metabolism; lipid metabolism; microbiota-generated metabolites) to either a personalized diet group or a control diet group (Figure 1) using a computer-generated sequence with random permuted block sizes of two within each stratum. Allocation to each cluster was based on analysis of metabolic and genetic biomarkers at baseline as described in detail elsewhere (23). The randomization was performed by a person not involved in the study to ensure blinding. Of the 100 adults recruited, 82 participants completed the 10-week intervention and—given the absence of differences between clusters (24)—were evaluated in a single-arm cohort in the present analysis (Figure 1). In addition, a subgroup analysis was performed as described below.

### Dietary intervention

All subjects received easy-to-prepare breakfast and dinner meal boxes twice a week (6 meals/delivery) for a total of 12 meals/week from the company Simple Feast (Copenhagen, Denmark); the diets were isocaloric and complied with national dietary guidelines on macronutrient distribution (23). The food provided was vegetarian and organically produced, however, participants were allowed to eat non-organic/non-vegetarian food as part of the meals not provided (lunches and all Saturday meals). Participants were instructed to consume the diets *ad libitum*. In contrast to the control group, meals for each cluster in the personalized diet group were supplemented with bioactive compounds provided by CARINSA (Spain). The food items, calorie and nutrient contents, and bioactive ingredients of the various meals have been presented elsewhere in detail (23). The meals of carbohydrate and microbiota clusters were both supplemented with an additional 20 g/d of fiber in the form of inulin and its hydrolyzed form fructooligosaccharide (FOS) extracted from chicory root as a functional ingredient, coupled with inulin-rich food items during the 10-week intervention (23). Therefore, these two clusters were pooled together into one subgroup for the purposes of this secondary exploratory analysis (Figure 1), referred to as ITF-prebiotics group (*n* = 21). Given that both the personalized and the control groups in the original study received high fiber (38 ± 11 g/d) plant-based meals with similar macronutrient composition, changes in outcomes (i.e., the difference between end-of-study and baseline measurements) were investigated in the whole cohort of completers (*n* = 82) and separately in the ITF-prebiotics subgroup (*n* = 21) and their controls (*n* = 22) (Figure 1).

Dietary intake was measured by 3-day estimated weight food records before and during week 3 of the 10-week intervention (two non-consecutive weekdays and one weekend day). Nutrient analysis was carried out with Vitakost (Conava ApS; Kolding, Denmark), which is based on the Danish national food composition database (frida.fooddata.dk, version 4, 2019).

Compliance was assessed twice a week with an electronic questionnaire, by reporting the proportion of food consumed from the provided meals during the previous 3 days (24).

### Anthropometrics and blood parameters

Assessment of anthropometrics (weight, height, waist circumference), body composition (by dual-energy X-ray absorptiometry) and blood pressure was conducted in the overnight fasted state. Furthermore, fasting blood samples were collected for the measurement of plasma glucose and serum triglyceride, total cholesterol (TC), low- and high-density lipoprotein cholesterol (LDL-C and HDL-C, respectively) and C-reactive protein (CRP) by using standard techniques at NEXS, University of Copenhagen with a Pentra 400 Analyzer (HORIBA ABX, France). Serum insulin was measured on the IMMULITE 2000 Immunoassay System (Siemens Healthcare Diagnostics Products Ltd., United Kingdom). Plasma interleukins 6 and 10 (IL6 and IL10, respectively), tumor necrosis factor α (TNFα) and monocyte chemoattractant protein 1 (MCP1) were determined by commercially available enzyme-linked immunosorbent assay (ELISA) according to the respective kit instructions.

### Gut microbiota analysis

Fecal samples were collected in a sterile container before and after the 10-week intervention and stored at −80°C until DNA extraction. The DNeasy PowerSoil Pro Kits (Qiagen, Germany) was used to extract total DNA from the fecal samples according to the manufacturer’s instructions. The final purified DNA was stored at −80°C. The bacterial DNA concentration was determined by using the Qubit HS Assay Kit (Invitrogen, Carlsbad, California, United States) on a Qubit 4 Fluorometric Quantification device (Invitrogen, Carlsbad, California, United States). The bacterial community composition was defined by using Illumina NextSeq-based high-throughput sequencing (HTS) (Illumina Inc.) of the 16S rRNA gene V3-region (25).

Raw sequence reads were imported into CLC genomic workbench (v8.5, CLCbio, Qiagen) as FASTQ files, demultiplexed and trimmed to remove barcodes and primers. The trimmed sequences were exported to Rstudio (version 4.0.5; Team RStudio 2015) and the Divisive Amplicon Denoising Algorithm 2 (DADA2) pipeline (v1.22) (26) was used to denoise and merge forward and reverse reads and generate Amplicon Sequence Variants (ASVs), which were filtered for chimeric sequences and the resulting ASV taxonomically classified using the Ribosomal Database Project database (27). In addition, for selected ASVs, nBLAST analysis (28) against the 16S rRNA gene database was performed in order to confirm taxonomy assigned according to the RDP database. The ASV taxonomic classification table and the ASV sequences and counts per sample were imported into Quantitative Insights into Microbial Ecology 2 software (QIIME 2 Core–2020.11) (29). ASVs assigned to Cyanobacteria/chloroplast or with a frequency <10 reads across all samples were removed and the core diversity metrics function was applied with a rarefaction depth of 11,000 reads per sample to generate α-diversity indices (Shannon index, Observed ASVs and Evenness index) and β- diversity (Bray-Curtis). Based on the filtered and rarefied ASV table, ASVs were collapsed into genus, family and phylum level taxonomy. Subjects with no detectable taxa at the given taxonomic level were given a value of half the detection limit (relative abundance of 0.000045).

### Quantification of serum lactate and branched-chain amino acids

Serum lactate and the branched-chain amino acids (BCAAs) leucine, isoleucine, and valine were quantified before and after the intervention using nuclear magnetic resonance (NMR), following extraction and concentration procedures as described previously (30). In brief, serum samples were placed in 2 mL 96-deepwell plates using 200 μL for aqueous extraction with methanol:water. After extraction, solvents from the samples were removed using a speed vacuum concentrator or N_2_ steam and then stored at −80°C until further analysis. For NMR measurements, the hydrophilic extracts were reconstituted in 600 μL of D_2_O phosphate buffer (PBS 0.05 mM, pH 7.4, 99.5% D_2_O) containing 0.73 mM trisilylpropionic acid (TSP). ^1^H NMR spectra were recorded at 300 K on an Avance III 600 spectrometer (Bruker, Germany) operating at a proton frequency of 600.20 MHz using a 5 mm PABBO gradient probe. One-dimensional ^1^H pulse experiments were carried out using the nuclear Overhauser effect spectroscopy (NOESY) presaturation sequence (RD–90°–t1–90°–tm–90° ACQ) to suppress the residual water peak, and the mixing time was set at 100 ms. The exponential line broadening applied before Fourier transformation was of 0.3 Hz. The frequency domain spectra were phased, baseline-corrected and referenced to TSP or TMS signal (d = 0 ppm) using TopSpin software (version 3.6, Bruker). All acquired ^1^H NMR from the PREVENTOMICS samples were compared to references of pure selected compounds with the metabolic profiling AMIX spectra database (Bruker), HMDB, and Chenomx databases for metabolite identification. After pre-processing, specific ^1^H NMR regions identified in the spectra were integrated using the AMIX 3.9 software package.

### Statistical analysis

Statistics were performed with QIIME2, R and SPSS Statistics (IBM SPSS Statistics, Version 28.0, IBM, Armonk, NY). Characteristics of study participants in the personalized and control groups at baseline were tested using *t*-test for normally distributed data, Mann–Whitney *U*-test for non–normally distributed data, or Pearson’s Chi-squared test for categorical data.

The change from baseline to week 10 in anthropometric measurements and health outcomes in all subjects (*n* = 82) were evaluated using paired *t*-test or Mann–Whitney U-test, as appropriate. One-way repeated measures ANOVA, adjusted for sex, was carried out for the changes from baseline within and between ITF-prebiotics (*n* = 21) and control (*n* = 22) subgroups. Changes in dietary intake extracted from food logs reported by the participants (valid data = 79; ITF-prebiotics = 20, Control = 22) were assessed with paired and independent *t*-tests for within the cohort and between the subgroups, respectively. Spearman’s rank correlation analysis was used to explore potential associations between significant changes in gut microbiota composition induced by ITF-prebiotics and host metabolic markers. Correlations between baseline log-transformed P/B ratios and changes in body weight were analyzed using Pearson’s correlation.

For gut microbiota analysis, the average of the top three most abundant phyla in the whole cohort as well as family and genus with an average relative abundance greater than 2% were included (Figure 3A). To compare the relative abundance between the ITF-prebiotics and control subgroups, a Mann–Whitney *U* test was performed, and Wilcoxon signed rank test was used for within group analyses. *p*-values of within group comparisons were corrected to control for false discovery rate (FDR) due to multiple testing according to the Benjamini and Hochberg procedure and a significance level of *q* < 0.05 was used. Beta diversity was examined with Bray-Curtis principal coordinates analysis (PCoA) scores from axis 1 and 2 as dependent variables to explore the change over time in the whole cohort as well as the differences between the two subgroups. All linear models were validated by investigating residual plots and q-q plots, and variables were log-transformed if required. *p*-values <0.05 were considered statistically significant.

## Results

### Study participants

In total, 82 subjects (26 men and 56 women) completed the study (24), of which 21 and 22 were assigned to the ITF-prebiotics and control subgroups, respectively (Figure 1). Baseline characteristics are presented in Table 1. More women than men were included but the sex distribution was not significantly different between the two subgroups (*p* = 0.23). There were no significant differences between the two subgroups in all outcomes at baseline.

**Table 1:** Characteristics of study participants at baseline.

	All (*n* = 82)	ITF-prebiotics (*n* = 21)	Control (*n* = 22)	*p*-value ITF-prebiotics vs. Control
Sex (% female)	56 (68%)	16 (76%)	13 (59%)	0.23
Age, years	45 ± 12	45 ± 10	42 ± 13	0.47
**Anthropometry**				
Body weight, kg	96 ± 16	96.7 ± 14	101 ± 15	0.31
Fat mass, kg	38.5 ± 9	40 ± 9.5	40 ± 9	0.95
Body mass index, kg/m^2^	32 ± 3.5	32.5 ± 3.4	33 ± 3.8	0.58
Waist circumference, cm	102 ± 11	104 ± 9.6	105 ± 11	0.79
Lean body mass, kg	54 ± 11	53 ± 10	57 ± 11	0.23
Visceral adipose tissue, g	1,427 ± 851	1,595 ± 847	1,631 ± 980	0.90
Total body fat, %	40 ± 7	41.5 ± 7	40 ± 7	0.49
**Blood Pressure**				
Systolic, mm Hg	122 ± 19	121 ± 15	126 ± 16	0.42
Diastolic, mm Hg	84 ± 10	85 ± 11	85 ± 10	0.98
**Glucose homeostasis**				
Glucose, mg/dL	94.0 ± 9.0	95.3 ± 8.7	95.2 ± 10.5	0.97
Insulin, mU/L	7.0 (4.7, 10.0)	8.2 (5.3, 12.1)	8.3 (5.4, 11.4)	0.88
HOMA-IR	1.5 (1.1, 2.4)	2.0 (1.1, 2.9)	1.9 (1.2, 2.7)	0.82
**Lipid Profile**				
Total cholesterol, mmol/L	4.9 ± 0.9	4.7 ± 0.8	4.9 ± 1.0	0.35
HDL cholesterol, mmol/L	1.4 ± 0.3	1.3 ± 0.3	1.24 ± 0.24	0.27
LDL cholesterol, mmol/L	3.0 ± 0.8	2.8 ± 0.7	3.3 ± 0.8	0.07
Triglycerides, mmol/L	1.0 (0.7,1.5)	1.1 (0.8, 1.6)	1.1 (0.8, 1.4)	0.89
Total cholesterol/HDL	3.7 ± 0.10	3.7 ± 0.95	4.1 ± 0.87	0.16
LDL/HDL	2.3 ± 89	2.2 ± 0.8	2.7 ± 0.8	0.06
**Inflammatory biomarkers**				
CRP, mg/L	1.4 (0.7, 2.8)	1.7 (0.8, 3.1)	1.3 (0.6, 2.3)	0.31
TNFα, pg./mL	0.50 ± 0.14	0.51 ± 0.09	0.49 ± 0.11	0.57
IL-6, pg./mL	1.2 (1.0, 1.9)	1.3 (1.0, 1.8)	1.1 (0.8, 1.5)	0.50
IL-10, pg./mL	1.0 (0.4, 1.3)	0.9 (0.4, 1.3)	1.0 (0.7, 1.3)	0.98
MCP1, pg./mL	175 ± 33	175 ± 37	182 ± 33	0.49
**Dietary intake**				
Energy, kcal/d	2,352 ± 654	2,303 ± 420	2,434 ± 777	0.50
Fat, %E	37 ± 7	35 ± 6	37 ± 8	0.36
Protein, %E	16 ± 3	16 ± 3	16 ± 4	0.90
Carbohydrate, %E	42 ± 8	45 ± 6	42 ± 8	0.13
Dietary fiber, g/d	26 ± 10	27 ± 7	22 ± 8	0.04

Data are presented as mean ± SD or median with quartiles, and comparisons between groups at baseline analyzed using the *t*-test for normally distributed data and the Mann–Whitney *U* test for non-normally distributed data. Categorical variables were analyzed by Pearson’s chi-square test. *p*-values are shown only for descriptive purposes and not for hypothesis testing.

The homeostatic model assessment of insulin resistance (HOMA-IR) was calculated as fasting plasma glucose (mmol/L) × fasting plasma insulin (mU/mL)/22.5.

ITF, inulin-type fructans; HDL, high-density lipoprotein; LDL, low-density lipoprotein; HOMA-IR, homeostatic model assessment of insulin resistance; CRP, C-reactive protein; TNFα, tumor necrosis factor α; IL6, interleukin 6; IL10, interleukin 10; MCP1, monocyte chemoattractant protein.

In the whole cohort, calorie intake significantly decreased (−280 ± 622 kcal/day), whereas fiber intake increased (12 ± 12.8 g/day) (both *p* < 0.001). The percent of the total energy from protein (−2.3 ± 3.0%) and fat (−2.3 ± 7.5%) decreased significantly (both *p* ≤ 0.01) while that from carbohydrate increased (4.3 ± 7.9%, *p* < 0.001). No statistically significant differences were observed between the control and ITF subgroups in the changes in energy and macronutrient intakes (all *p* > 0.10) except for fiber intake which increased in both but significantly more in the ITF than in the control group (by 24 ± 12 and by 10.5 ± 9.9 g/day, respectively, *p* < 0.001).

The achieved level of compliance was high for all participants and subgroups, with an average of 90 ± 11% of the delivered meals being consumed during the 10-week intervention period (24).

### Plant-based diet, with or without isolated ITF-prebiotics, improves health markers

Participants in the whole cohort reduced their weight (−3.2 kg ± 3.1, *p* < 0.001), waist circumference (−2.2 ± 3.3 cm, *p* < 0.001), and fat mass (−2.0 ± 2.3 kg, *p* < 0.001), and experienced significant improvements in glucose homeostasis with no differences between diet subgroups (Table 2). Improvements in lipid profile were observed in the whole cohort, however, a significant reduction in LDL-and total cholesterol over time was observed only among the control subgroup, whereas a significant reduction in HDL-C was observed only in the ITF-prebiotics subgroup. Accordingly, the LDL:HDL ratio was significantly lower in the control compared to ITF subgroup at the end of the study, despite similar weight loss.

**Table 2:** Effect of plant-based diet and added ITF-prebiotics on the change of anthropometric measurements and health parameters.

	All (*n* = 82)^a^	ITF-prebiotics (*n* = 21)	Control (*n* = 22)	*p*-value (ITF-prebiotics vs. Control)
**Anthropometry**				
Body weight, kg	−3.2 ± 3.1^**^	−3.3 ± 0.7^**^	−3.7 ± 0.7^**^	0.70
Body mass index, kg/m^2^	−1.0 ± −1.0^**^	−1.1 ± 0.2^**^	−1.2 ± 0.2^**^	0.86
Waist circumference, cm	−2.2 ± 3.3^**^	−2.7 ± 0.8^**^	−2.6 ± 0.8^**^	0.97
Lean body mass, kg	−1.0 ± 1.3^**^	−0.9 ± 0.3^**^	−1.3 ± 0.3^**^	0.33
Fat mass, kg	−2.0 ± 2.3^**^	−2.3 ± 0.6^**^	−2.4 ± 0.6^**^	0.96
Visceral adipose tissue, g	−143 ± 236^**^	−193 ± 57^**^	−170 ± 56^**^	0.78
Total body fat, %	−1.0 ± 1.3^**^	−1.0 ± 0.4^**^	−1.0 ± 0.4^**^	0.99
**Blood Pressure**				
Systolic, mm Hg	0.2 ± 15	0.2 ± 3.0	−2.0 ± 3.0	0.62
Diastolic, mm Hg	−3 ± 6.6^**^	−2.7 ± 1.1^*^	−3.3 ± 1.1^**^	0.72
**Glucose homeostasis**				
Glucose, mg/dL	−1.4 ± 5.8^*^	−0.2 ± 1.3	−0.9 ± 1.3	0.73
Insulin, mU/L^**b**^	−10 (−26, 5)^**^	−10 (−21, 3)	−11 (−22, 1)	0.91
HOMA-IR^**b**^	−10.5 (−29, 3)^**^	−10 (−22, 4)	−11 (−23, 2)	0.88
**Lipid Profile**				
Total cholesterol, mmol/L	−0.3 ± 0.5^**^	−0.2 ± 1.0	−0.3 ± 1.0^**^	0.29
HDL cholesterol, mmol/L	−0.06 ± 0.15^**^	−0.10 ± 0.03^**^	−0.05 ± 0.03	0.23
LDL cholesterol, mmol/L	−0.16 ± 0.44^**^	−0.04 ± 0.08	−0.24 ± 0.07^**^	0.07
Triglycerides, mmol/L^**b**^	−4.4 (−23, 31)	1.4 (−13, 19)	3.3 (−11, 20)	0.88
Total cholesterol/HDL	−0.06 ± 0.50	0.15 ± 0.11	−0.13 ± 0.11	0.07
LDL/HDL	−0.04 ± 0.42	0.16 ± 0.09	−0.10 ± 0.09	**0.04**
**Inflammatory biomarkers**				
CRP, mg/L^**b**^^,^^**c**^	−6.7 (−41, 42)	−1.6 (−31, 41)	−1.4 (−30, 39)	0.99
TNFα, pg./mL	0.02 ± 0.07^*^	0.05 ± 0.02^**^	0.01 ± 0.02	0.07
IL-6, pg./mL^**b**^	2.5 (−24, 27)	−1.1 (−17, 19)	9 (−9, 31)	0.43
IL-10, pg./mL^**b**^	9.6 (−6, 39)^**^	51 (14, 102)^**^	19 (−10, 58)	0.24
MCP1, pg./mL	4.2 ± 21	10.9 ± 5.3^*^	4.7 ± 5.2	0.43

a Data for all participants are presented as mean ± SD or median percent change with (quartiles), using paired *t*-test for normally distributed data and the Mann–Whitney *U* test for non-normally distributed data. To observe differences between the basal state and week-10 as well as between ITF-prebiotics and Control, one-way repeated measure ANOVA, adjusted for sex, was performed and values are presented as estimated mean ± SEM.

b Log-transformation was performed to non-normally distributed residuals, and data thus represent mean percent change with its corresponding 95% CI.

c Excluding 1 outlier (*n* = 1).

^**^p < 0.01, ^*^p < 0.05 significant difference from baseline. Significant difference between the subgroups are displayed in bold.

The homeostatic model assessment of insulin resistance (HOMA-IR) was calculated as fasting plasma glucose (mmol/L) × fasting plasma insulin (mU/mL)/22.5.

ITF, inulin-type fructans; HDL, high-density lipoprotein; LDL, low-density lipoprotein; HOMA-IR, homeostatic model assessment of insulin resistance; CRP, C-reactive protein; TNFα, tumor necrosis factor α; IL6, interleukin 6; IL10, interleukin 10; MCP1, monocyte chemoattractant protein 1.

With respect to inflammatory markers, significant increases in TNFα and IL-10 in the whole cohort were driven largely by the ITF-prebiotics subgroup. MCP-1 also increased in the ITF-prebiotics group but not in the control group. Overall, however, no significant differences were observed between subgroups (Table 2).

### Inulin-type fructans reduces the number of observed species as well as the Shannon diversity index in the ITF-subgroup

**Figure 2:**
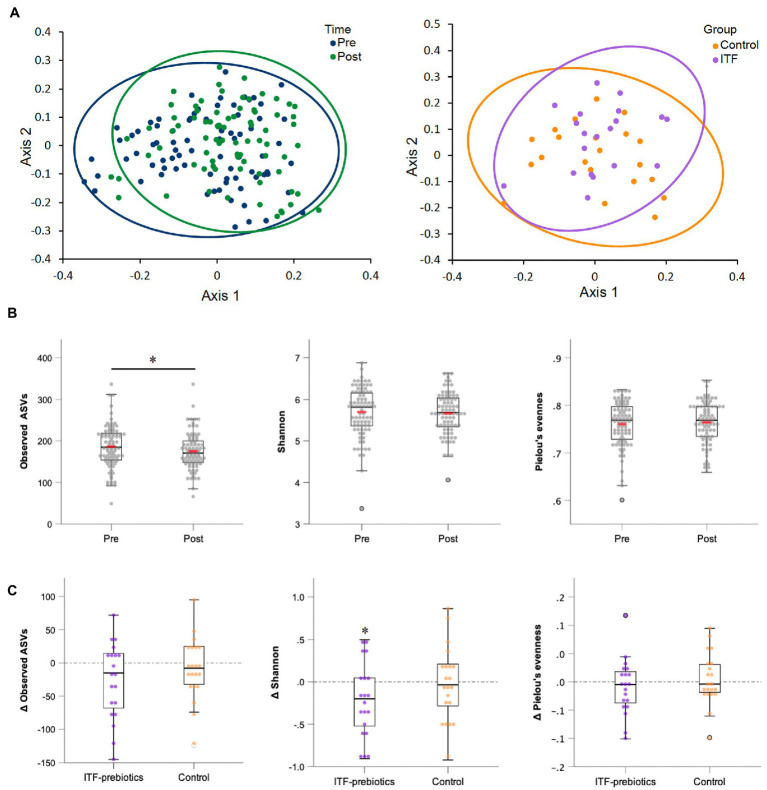
Alpha-and beta-diversity comparisons of the gut microbiome from baseline to end-of-trial. **(A)** The change in principal coordinate analysis (PCoA) of the β-diversity index Bray Curtis (*p* < 0.01) in all participants (*n* = 80) (on the left), and between ITF-prebiotics (*n* = 20) vs. control (*n* = 21) (on the right) after the dietary intervention (*p* = 0.21). **(B)** Changes in α-diversity indices: number of observed species (*p* = 0.03), Shannon (*p* = 0.26) and Pielou’s evenness (*p* = 0.67) in all participants (*n* = 80), and in **(C)** ITF-prebiotics (*n* = 20) vs. control (*n* = 21) from baseline. ^*^p < 0.05, ^**^p < 0.01. ITF, inulin-type fructans.

Two stool samples were excluded from analysis after quality control due to unexpected low reads (<3,000), resulting in 80 out of the collected 82 fecal samples being included in the analysis (Table 3). Metrics of α-diversity (number of observed species, Shannon and Pielou’s evenness) showed that the whole cohort (*n* = 80) had a significant decrease in richness (*p* = 0.034) (Figure 2B), mainly driven by ITF supplementation (ITF -26.3± 57, *p* = 0.05 vs. control -8.4± 46.4, *p* = 0.42; Figure 2C), which also decreased Shannon diversity (*p* = 0.035) (Figure 2C). The increase in α-diversity (Shannon index) correlated with improvements in insulin sensitivity and with reductions in visceral adipose tissue and fat mass in the ITF-subgroup (*p* < 0.05). Principal coordinate analysis (PCoA) based on Bray Curtis dissimilarity metrics detected systematic change from baseline to end-of-trial in the whole cohort (*n* = 80) but no differences between the two subgroups (Figure 2A).

**Table 3:** Changes in relative abundance of fecal bacteria at the family, phylum and genus level in subjects receiving plat-based diet for 10 weeks.

	All (*n* = 80)		ITF-prebiotics^a^ (*n* = 20)		Control (*n* = 21)		*p*-values^b^		
	Pre	Post	Pre	Post	Pre	Post	All	ITF-prebiotics	Control
**Phyla**									
*Firmicutes*	0.745 ± 0.108	0.724 ± 0.116	0.763 ± 0.071	0.728 ± 0.112	0.749 ± 0.096	0.705 ± 0.136	0.13	0.23	0.65
*Bacteroidetes*	0.140 ± 0.113	0.139 ± 0.106	0.131 ± 0.087	0.123 ± 0.085	0.123 ± 0.103	0.135 ± 0.118	0.65	0.68	0.36
*Actinobacteria*	0.084 ± 0.067	0.098 ± 0.065	0.081 ± 0.060	0.124 ± 0.063	0.101 ± 0.067	0.114 ± 0.065	0.90	**0.02**	0.41
**Family**									
*Lachnospiraceae*	0.372 ± 0.110	0.344 ± 0.092	0.383 ± 0.081	0.327 ± 0.092	0.368 ± 0.090	0.329 ± 0.094	**0.03**	**0.03**	0.26
*Ruminococcaceae*	0.230 ± 0.072	0.254 ± 0.074	0.231 ± 0.067	0.275 ± 0.080	0.238 ± 0.058	0.250 ± 0.060	**0.01**	**0.04**	0.46
*Bacteroidaceae*	0.065 ± 0.067	0.065 ± 0.069	0.075 ± 0.070	0.074 ± 0.075	0.049 ± 0.054	0.052 ± 0.043	0.91	1.00	0.43
*Bifidobacteriaceae*	0.049 ± 0.061	0.071 ± 0.063	0.045 ± 0.052	0.097 ± 0.064	0.064 ± 0.059	0.085 ± 0.064	**<0.01**	**0.01**	0.32
*Erysipelotrichaceae*	0.048 ± 0.047	0.036 ± 0.032	0.052 ± 0.063	0.033 ± 0.039	0.045 ± 0.042	0.032 ± 0.025	**0.02**	0.13	0.34
*Prevotellaceae*	0.038 ± 0.074	0.041 ± 0.069	0.016 ± 0.033	0.021 ± 0.036	0.045 ± 0.073	0.047 ± 0.083	0.91	0.18	0.27
*Coriobacteriaceae*	0.026 ± 0.020	0.019 ± 0.014	0.029 ± 0.021	0.022 ± 0.012	0.028 ± 0.029	0.019 ± 0.016	**<0.01**	0.10	0.20
**Genera**									
*Faecalibacterium*	0.121 ± 0.069	0.154 ± 0.072	0.128 ± 0.061	0.186 ± 0.082	0.126 ± 0.052	0.147 ± 0.052	**<0.01**	**0.04**	0.52
*Lachnospiraceae, unclassified genus*	0.101 ± 0.033	0.096 ± 0.030	0.108 ± 0.049	0.086 ± 0.030	0.092 ± 0.026	0.089 ± 0.027	0.50	0.28	0.80
*Blautia*	0.099 ± 0.058	0.084 ± 0.048	0.103 ± 0.054	0.071 ± 0.04	0.104 ± 0.051	0.096 ± 0.059	**0.04**	0.10	0.73
*Bifidobacterium*	0.049 ± 0.061	0.071 ± 0.064	0.045 ± 0.053	0.097 ± 0.064	0.064 ± 0.059	0.085 ± 0.064	**<0.01**	**0.03**	0.40
*Agathobacter*	0.041 ± 0.042	0.033 ± 0.028	0.039 ± 0.032	0.031 ± 0.023	0.040 ± 0.040	0.026 ± 0.023	0.50	0.76	0.45
*Bacteroides*	0.036 ± 0.043	0.034 ± 0.038	0.053 ± 0.055	0.044 ± 0.045	0.021 ± 0.015	0.025 ± 0.021	0.78	0.71	0.66
*Prevotella*	0.031 ± 0.068	0.036 ± 0.063	0.015 ± 0.032	0.016 ± 0.032	0.035 ± 0.070	0.041 ± 0.075	0.48	0.30	0.69
*Ruminococcaceae, unclassified genus*	0.034 ± 0.026	0.031 ± 0.021	0.037 ± 0.031	0.033 ± 0.025	0.032 ± 0.023	0.030 ± 0.19	0.41	0.64	0.76
*Gemmiger*	0.033 ± 0.025	0.031 ± 0.026	0.028 ± 0.018	0.025 ± 0.019	0.040 ± 0.034	0.037 ± 0.025	0.50	0.73	0.66
*Phocaeicola*	0.028 ± 0.035	0.030 ± 0.037	0.020 ± 0.020	0.029 ± 0.038	0.028 ± 0.043	0.026 ± 0.030	0.50	0.43	0.80
*Clostridium_XlVa*	0.026 ± 0.018	0.023 ± 0.017	0.033 ± 0.019	0.032 ± 0.023	0.027 ± 0.021	0.023 ± 0.016	0.47	0.95	0.75
*Collinsella*	0.025 ± 0.020	0.018 ± 0.013	0.028 ± 0.021	0.021 ± 0.012	0.025 ± 0.027	0.018 ± 0.016	**0.01**	0.30	0.52
*Roseburia*	0.020 ± 0.026	0.023 ± 0.022	0.019 ± 0.021	0.022 ± 0.023	0.016 ± 0.021	0.016 ± 0.020	0.44	0.92	0.97

a Group receiving 20 g/d inulin + fructooligosaccharides.

b *p*-values were adjusted to control the false discovery rate for multiple testing according to the Benjamini and Hochberg procedure. Significant changes are displayed in bold.

Values are displayed as means ± standard deviation using Wilcoxon paired test. ITF, inulin-type fructans.

### Intervention-induced changes in gut microbiota taxa

In the whole cohort, the relative abundance between baseline and end-of-trial was increased for *Ruminococcaceae* and *Bifidobacteriaceae* (0.024 ± 0.075, *P_FDR =_* 0.01; and 0.022 ± 0.062, *P_FDR_* < 0.01; respectively, Table 3) and decreased for *Lachnospiraceae* (−0.03 ± 0.11, *P_FDR_* < 0.03). These changes were largely driven by ITF supplementation (Table 3). Furthermore, the relative abundance of *Erysipelotrichaceae* (−0.012 ± 0.034, *P_FDR_* = 0.02) and *Coriobacteriaceae* (−0.007 ± 0.020, *P_FDR_* < 0.01) was reduced in the whole cohort.

**Figure 3:**
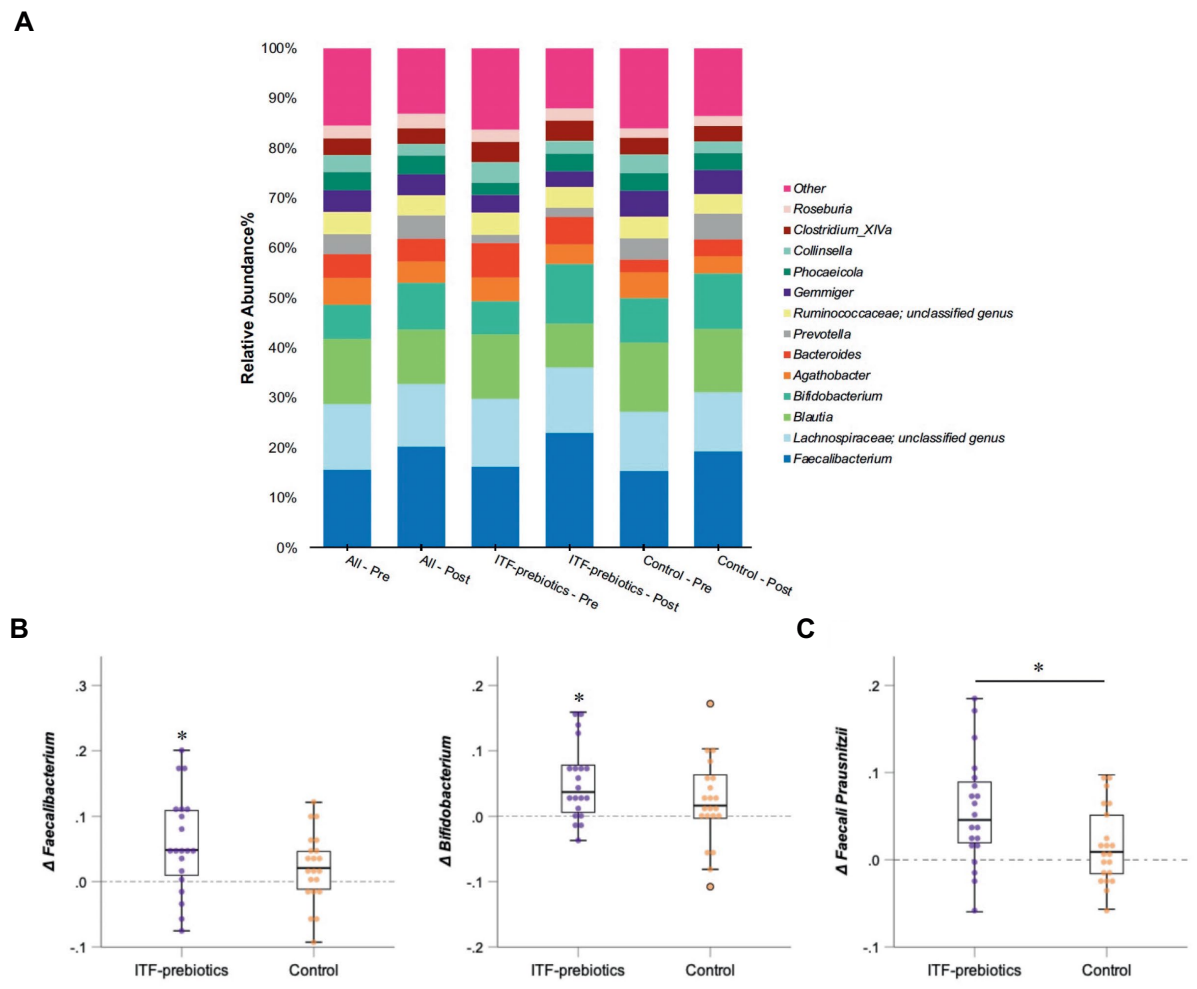
Changes in microbial composition from baseline to end of trial. **(A)** Relative abundances of bacterial taxa accounting for more than 2% at the genus level assessed using Illumina 16S rRNA gene sequencing in subjects with obesity (all cohort, *n* = 80) consuming plant-based diet and in a subgroup consuming ITF-prebiotics (*n* = 20) vs. their controls (*n* = 21). **(B)** Genera and **(C)** species with significant changes from baseline in ITF-prebiotics group analyzed using Wilcoxon paired test with a false discovery correction according to the Benjamini and Hochberg procedure. Mann–Whitney *U* test was performed to compare the relative abundance between the control and ITF-prebiotics subgroups. ^*^p < 0.05, ^**^p < 0.01 significant change from baseline. ITF, inulin-type fructans.

There was a significant increase in *Actinobacteria* phylum in the ITF-prebiotics group (0.043 ± 0.061, *P_FDR_* = 0.02). At the genus level, supplementation with ITF-prebiotics resulted in a significant increase in the relative abundance of *Bifidobacterium* (0.052 ± 0.058, *P_FDR_* = 0.03) and *Faecalibacterium* (0.058 ± 0.075 *P_FDR_* = 0.04) (Figure 3B); the latter seemed to be increased at the expense of *Collinsella* in the ITF-subgroup (*r* = −0.5, *p* < 0.02) and in all subjects (*n* = 80, *r_s_* = −0.4, *p* < 0.001). Notably, the change in *Faecalibacterium* abundance following ITF supplementation correlated positively with the change in insulin and HOMA-IR score (*r_s_* = 0.59, *p* < 0.01; *r_s_* = 0.57, *p* < 0.01) and negatively with HDL-C (*r_s_* = −0.5, *p* = 0.02). In addition, the change in *Bifidobacterium* significantly correlated with the reduction in total cholesterol (*r_s_* = −0.5, *p* = 0.01) and LDL-C (*r_s_* = −0.5, *p* = 0.04) in the control group. A significant increase in the relative abundance of *Faecalibacterium prausnitzii (F. prausnitzi)* species was detected in the ITF-prebiotics subgroup (Figure 3C). All changes from baseline to end-of-trial in the control group were not significant, and no significant differences between subgroups were observed, except for the change in *F. prausnitzii* (Figure 3C).

### Weight loss is Not predicted by baseline fecal P/B

**Figure 4:**
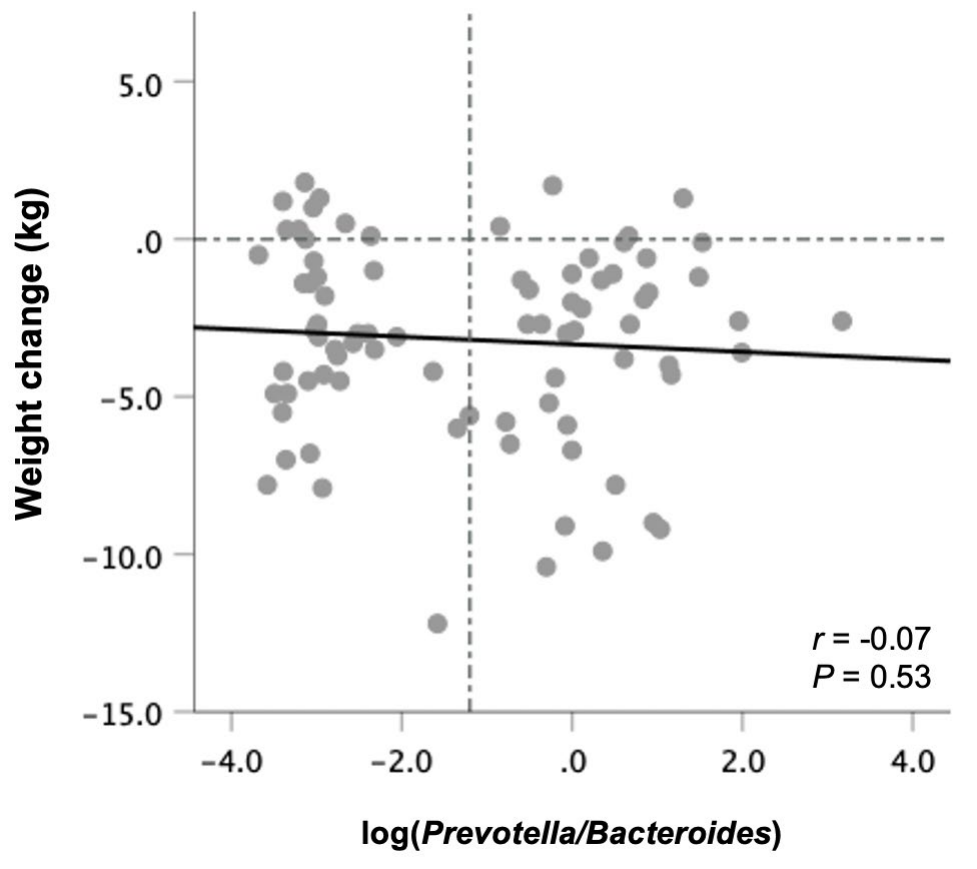
Changes in body weight from baseline to end-of-trial in relation to baseline fecal log-transformed *Prevotella*-to-*Bacteroides* ratio in all participants (*n* = 82). Pearson’s correlation coefficients and *p* values are displayed. Vertical line denotes the mean log *P/B* ratio.

*Prevotella* was detected in 40 subjects at baseline, whereas 42 had non-detectable *Prevotella* levels and were assigned a value of half the limit of detection (relative abundance of 0.000045). There was no correlation between the P/B ratio at baseline with weight changes following the 10-week study period in all participants (Figure 4) or those in the ITF and control subgroups. The same was true when excluding subjects with non-detectable *Prevotella* from the analysis.

### Effect of the dietary intervention on lactate and BCAAs

As expected, serum lactate and BCAAs were significantly (all *p* < 0.01), and to the same extent, reduced in both subgroups given the consumption of very similar plant-based diets over the 10-week study period.

## Discussion

The present study explored changes in gut microbiota and host metabolism after 10 weeks on a high fiber plant-based diet with or without the addition of isolated ITF-prebiotics. Our results reinforce current evidence demonstrating that plant-based diets are effective in reducing body weight and improving glucose and lipid profiles (7–9). We found that a predominantly plant-based diet (with a fiber content of ∼38 g/d) consumed *ad libitum* for a period of 10 weeks reduced fasting glucose, insulin, total cholesterol, and LDL-C levels, concomitant with improvements in measurements of body composition including fat mass, body fat percentage, total and visceral adipose tissue, and waist circumference. However, the addition of 20 g/d isolated ITF-prebiotics to the already high fiber diet did not provide additional benefits on various physiological parameters. If anything, ITF supplementation mitigated some of the beneficial effects of the plant-based diet on cardiometabolic risk factor profile, for the same amount of weight loss. This is in line with previous studies that found supplementation with inulin and/or FOS does not have significant independent effects on fasting glycemia and insulinemia, HOMA-IR index or lipid profile (31–35). Nonetheless, others demonstrated that ITF-prebiotics may have lipid-lowering (36) or glucose-lowering (13, 37, 38) effects, particularly among those with impaired fasting glycemia or hypercholesterolemia. In the present study we recruited individuals with obesity who were otherwise healthy (i.e., without hypertension, glucose abnormalities, and dyslipidemia), and found trends for attenuated reduction in glucose and LDL-C and augmented reduction in HDL-C in the ITF-subgroup relative to control, despite similar weight loss. These observations raise the possibility that the glucose and lipid-modifying ability of ITF-prebiotics mostly benefits those with compromised glycemic control and lipid profile at baseline. From a mechanistic perspective, a review by McRorie et al. (39) proposed that the cholesterol-lowering and improved glycemic effects of soluble fibers are highly correlated with their high viscosity gel-forming properties. Accordingly, non-viscous soluble fibers such as inulin and FOS do not exhibit these viscosity/gel-dependent beneficial health effects (39, 40).

Supplementation with ITF-prebiotics for 10 weeks altered the fecal bacterial community of the participants. Overall, in agreement with previous findings (38), we observed a decrease in gut microbiota richness among the whole cohort, which was mainly driven by the ITF-prebiotics subgroup (reduced alpha diversity measures). Therefore, it may be inferred that providing one type of isolated fiber for enough time induces a decrease in diversity and promotes the growth of certain taxa attributable to their specific ability to metabolize this type of fiber. Of particular interest, the administration of 20 g/d ITF-prebiotics in the present study led to a selective increase in the abundances of *Bifidobacterium* (∼2.2 fold) and *F. prausnitzii* (∼1.6 fold), in line with previous studies (31, 41–44). Specific stimulation of these taxa as a function of prebiotics has been shown to be associated with various health parameters resulting from cross-feeding interactions between acetate-producing bifidobacteria with butyrate-producing *F. prausnitzii* (45). Despite the bifidogenic effect of ITF in the present study, no strong correlation was found between changes in *Bifidobacterium* and *F. prausnitzii* abundances with changes in glucose and triglyceride concentrations, as reported previously (46). However, *Faecalibacterium species* abundance was positively correlated with serum insulin levels and HOMA-IR, contrary to previous findings (47). Interestingly, a recent study analyzing *Faecalibacterium*-like metagenome-assembled genomes identified twelve clades of this genus in the human gut, suggesting different functional potential and diversity associated with diet, health, and disease (48). That said, investigating which clade or strain is associated with a particular function is yet to be explored.

Obesity and T2D are recognized as states of chronic low-grade inflammation associated with gut dysbiosis and manifested by higher levels of circulating proinflammatory cytokines (3). Current evidence on the potential influence of gut microbes on immune responses are largely derived from *in vitro* and animal models, whereas findings from human studies are few and equivocal. Several studies have reported that the microbial diversity and the abundance of *Bifidobacterium* species and butyrate-producing bacteria, e.g., *F. prausnitzii,* in the human colon is markedly decreased in patients with gut diseases such as inflammatory bowel disease (49). *F. prausnitzii* are involved in immunomodulation by downregulating the NF-κB pathway and consequently inhibiting the synthesis of pro-inflammatory cytokines IL-6 and IL-12, while facilitating the induction of anti-inflammatory IL-10 (50). An *in vitro* study by Pang et al. demonstrated that inulin-type fructans, derived from Platycodon grandifloras, induced a significant increase in IL-10 mRNA levels along with a minor increase in TNFα (51). Another study confirmed that FOS and inulin independently increased TNFα, IL-1β, and IL-10 in human peripheral blood monocytes mediated through TLR signaling (52). In fact, only TNFα and IL-10 were significantly induced (52). This direct role was investigated *in vitro* demonstrating that ITF-prebiotics act as TLR4 ligands in intestinal epithelial cells and induce the production of MCP-1 and macrophage inflammatory protein 2 (MIP2), with an efficacy that was 50–80% that of lipopolysaccharide (53). Our study yielded similar results with ITF supplementation significantly increasing IL-10 and MCP-1 levels and mildly increasing TNFα. On the other hand, Clarke et al. reported a decrease in serum IL-10 concentration following 28 days of 15 g/d fructans consumption among 30 healthy adults (54), and Dehghan et al. found a decrease in TNFα, CRP and lipopolysaccharide among women with T2D after 8 weeks of 10 g/d inulin supplementation together with a trend for increased IL-10 (55). The reasons for the conflicting results observed across studies remain to be clarified. Interestingly, however, Hippe et al. (56) reported that *F. prausnitzii* phylotypes found in individuals with obesity and T2D were different compared to those found in lean counterparts who had the highest in *F. prausnitzii* but the lowest in its butyrate production capacity (BUT gene). Notably, the highest BUT gene abundance was observed among patients with T2D, suggesting that butyrate-producing capacity differs between *F. prausnitzii* phylotypes, and its protective role may be concentration-dependent. The authors speculated that a specific threshold of butyrate production can be protective—improving adiposity and glucose homeostasis; whereas increased concentrations could be detrimental—promoting inflammation. There is still much to be learned by investigating the complex signaling pathways involved in the direct and indirect prebiotics-gut microbiota interactions with host health including the immune system, leading to either an anti-or a pro-inflammatory response.

With respect to weight management in relation to microbial enterotypes, in contrast to previous findings (20, 21), baseline P/B ratio in this study did not predict weight loss success. However, in accordance with previous observations (19, 22), the association between the *Prevotella* enterotype and weight loss was observed only among a subgroup of individuals having low salivary amylase gene copy number. Here, we did not evaluate this gene variant. Also, about half of the participants (51%) had no detectable fecal *Prevotella* at baseline. This is higher than previously reported (19–21) and basically excluded half of the participants from the analysis, resulting in low statistical power for detecting significant relationships with health outcomes. Moreover, previous research suggests that individuals with no detectable *Prevotella* have a differential weight loss response compared to those with low P/B ratio (20).

To our knowledge, this is the first study that compared the health effects of consuming a diet naturally rich in fiber, with prebiotic properties, and its effects on gut microbiota composition and physiological outcomes relative to a similar plant-based diet supplemented with inulin and FOS. We demonstrate that improvements in various health parameters can be achieved with a varied high-fiber diet rather than supplementation with an isolated nutrient which in fact was associated with reduced microbial diversity and attenuated cardiometabolic benefits. However, these results should be interpreted with caution given the exploratory nature of our analysis. Intriguingly, other studies (57, 58) reported favorable changes in metabolic markers following dietary intervention with ITF-prebiotic despite observed reduced gut microbiota diversity, which appear to contradict the notion that overall greater diversity and/or richness translates to better health. Therefore, the relationships between decreased gut microbial diversity and health outcomes in addition to what constitutes a healthy gut microbiome require further investigation. In addition, we did not measure bowel habits or gastrointestinal symptoms but we nevertheless encountered only 4 dropouts in the ITF-prebiotics subgroup. It has previously been reported that inulin fructans are generally well-tolerated up to a level of 20 g/d (59). An important limitation concerning research with gut microbiome is the lack of quantitative data on actual fluxes of SCFAs and of relevant metabolic processes and interconversions resulting from prebiotics fermentation. Similarly, our study provides no insight into gut microbial SCFA production. Considering that the majority of SCFAs are rapidly utilized by colonocytes (butyrate being the primary energy source), or consumed in cross-feeding between different gut microbiota (leaving only ∼5% of total SCFAs excreted in stool), interpretation of concentrations in peripheral blood and/or urine may not accurately reflect the amounts produced in the gut and subsequently absorbed in the systemic circulation. Lastly, it is possible that the absence of significant differences between the two subgroups in our study was due to the control diet being already very high in fiber (33 g/d) and plant foods; inulin is naturally present in a wide variety of plants such as onions, garlic, leek, bananas, asparagus, artichoke, wheat, and chicory (60).

Collectively, while evidence for direct and indirect health effects of prebiotics is rapidly accumulating, results from interventions in human subjects are inconsistent and make it challenging to draw comprehensive conclusions, thereby limiting translation to practice. This could be related to (1) the heterogeneity of study designs and the generally small sample sizes, (2) the habitual intake of dietary fiber, (3) the duration of supplementation as well as factors related to differences in the type, dose, and physicochemical properties of the prebiotics, and (4) the gut microbiota’s fermentation capacity. The bidirectional relationship between gut microbiota and host adds further complexity. Therefore, future research efforts should focus on unraveling the potential mechanisms underlying these complex pathways and microbial functionality through metagenomic analysis before targeted dietary advice can be implemented. Well-powered large-scale prospective randomized multi-omics trials designed to tailor a diet based on baseline microbiota profile, amongst others, are warranted to provide more concrete evidence. Consideration of individual factors, including, but not limited to, health status, age and sex could also explain the variation of the microbiota responses to diet among individuals.

## Conclusion

In summary, our results indicate that *ad libitum* consumption of a balanced plant-based diet that is naturally rich in fiber significantly decreases body weight and improves glycemic and lipid profiles, whereas the addition of ∼20 g/d of isolated inulin-type fructans has no additional benefits in generally healthy individuals with obesity despite modulating gut microbiota composition. There is insufficient evidence at present to support precision dietary advice for weight loss merely informed by individual gut microbiota profile.

## Data availability statement

The original contributions presented in the study are publicly available. This data can be found at: https://www.ncbi.nlm.nih.gov/sra/PRJNA908225.

## Ethics statement

The studies involving human participants were reviewed and approved by the ethical committee of the Capital Region of Denmark (H-20029882). The patients/participants provided their written informed consent to participate in this study.

## Author contributions

MA, KP, MH, and FM: designed and conducted the original trial. MA, LC, MH, and FM: conceived the idea and designed the current analysis. XM and ML: carried gut microbiota characterization analyses. MA and KP: undertook data acquisition of the original study. MA: conducted data analysis and acquired fund for gut microbiome analyses. MA and FM wrote the original draft. MA, XM, ML, KP, LC, HR, DN, MH, and FM critically revised and edited the manuscript. All authors contributed to the article and approved the submitted version.

## Funding

The PREVENTOMICS project was supported by the European Union’s Horizon 2020 research and innovation program under grant agreement no. 818318, but no additional funding was received for the current analysis. Work on gut microbiome quantification in feces in addition to some of the study materials was funded by a PhD scholarship from King Saud bin Abdulaziz University for Health Sciences *via* The Saudi Arabian Cultural Office.

## Conflict of interest

The authors declare that the research was conducted in the absence of any commercial or financial relationships that could be construed as a potential conflict of interest.

## Publisher’s note

All claims expressed in this article are solely those of the authors and do not necessarily represent those of their affiliated organizations, or those of the publisher, the editors and the reviewers. Any product that may be evaluated in this article, or claim that may be made by its manufacturer, is not guaranteed or endorsed by the publisher.
